# A Single-Center Experience of Internal Pancreatic Fistulas

**DOI:** 10.7759/cureus.29181

**Published:** 2022-09-15

**Authors:** A Siva Sankar, Prakashen O K, Khamar J Banu, M Pon Chidambaram

**Affiliations:** 1 Surgical Gastroenterology, Government Mohan Kumaramangalam Medical College Hospital, Salem, IND; 2 Surgical Gastroenterology, Madras Medical College, Chennai, IND

**Keywords:** chronic pancreatitis, early surgical intervention, pancreatic pleural effusion, pancreatic ascites, internal pancreatic fistula

## Abstract

Background

Internal pancreatic fistula (IPF) is a complex disease with different etiologies, varied clinical presentations, and multiple management options. Unlike postoperative pancreatic fistula, IPF lacks guidelines for classification and management. The rarity of the disease makes randomized control studies unlikely and difficult to formulate guidelines. This has resulted in different approaches to managing IPF. IPF associated with both acute and chronic pancreatitis is treated with a step-up approach. Chronic pancreatitis-associated IPF treated with the traditional step-up approach is associated with increased morbidity. Prolonged fasting, drainage of protein-rich pancreatic fluid, and extended hospital stay add to the morbidity. Early surgical intervention in patients with IPF associated with chronic pancreatitis can treat both the fistula and underlying disease processes simultaneously. This may contribute to reduced morbidity and hospital stay.

Methodology

A retrospective observational study was conducted between June 2018 and May 2019. IPF patients with fluid amylase >1,000 IU/L and fluid albumin >3 g/dL were included in the study.

Results

In total, 32 patients were included in the study. A total of 13 patients had acute pancreatitis and 19 were associated with chronic pancreatitis. Pseudocyst and walled-off pancreatic necrosis were present in 18 patients. The duration of treatment for the traditional group was 8-14 weeks, and for the early surgery group, it was 8-10 days. Patients were followed up for two years, and none of the patients in the early surgery group had a recurrence.

Conclusions

The overall mortality of IPF is low but it has high morbidity. The delay in treatment may contribute to high morbidity; hence, early surgical intervention may change the clinical course. The primary pathology of the pancreas can be addressed simultaneously as well. In our study, early surgical intervention was associated with lesser morbidity and decreased duration of hospital stay while recurrence rates and mortality were comparable to the traditional management protocol.

## Introduction

Pancreatic fistulas can occur as a complication of acute or chronic pancreatitis. Trauma or iatrogenic injury to the duct following surgery of the pancreas or nearby organs, endoscopic intervention, or percutaneous procedures can also result in pancreatic fistulas. Unlike postoperative external pancreatic fistulas, there are no adequate guidelines for the classification and management of internal pancreatic fistulas (IPFs). IPFs can manifest as pancreatic ascites, pancreatic pleural effusion, mediastinal effusion, bronchial effusion, pancreaticocardial [1] or pancreaticoenteric fistulas, and, on rare occasions, pancreaticobiliary [2] or pancreaticoportal fistulas [3].

The common underlying mechanism for fistula formation is pancreatic ductal disruption. Internal fistulas can develop from an uncontained disruption of the pancreatic duct, resulting in a leakage of pancreatic fluid into the pleural, peritoneal, or mediastinal cavities. Less commonly, IPFs can occur from a rupture or leak of a pseudocyst or walled-off pancreatic necrosis (WOPN). An IPF is seen in 7-8% of chronic pancreatitis cases [4] and in approximately 1% of acute pancreatitis cases.

Anterior ductal disruption can cause pancreatic ascites, while posterior ductal disruption can cause pleural effusion, predominantly on the left side. IPFs should be suspected in any patient with acute or chronic pancreatitis with ascites or massive pleural effusion. The nature of the fluid is confirmed by elevated levels of amylase (>1,000 IU/L) and albumin (>3 g/dL).

Management of IPFs depends on the acute or chronic nature of the disease, ductal anatomy, and associated pseudocysts. Initial nonoperative management can lead to symptom resolution in 40-60% of patients. If the patient remains stable, conservative management can be continued for three weeks [5]. Failure of conservative management, evidenced by recurrence or the onset of new symptoms such as respiratory distress due to tense ascites or pleural effusion, is an indication for drainage. Failure of percutaneous treatment may be due to proximal ductal obstruction by stones, strictures, and/or disconnected ducts. Endoscopic retrograde cholangiopancreatography (ERCP) is used for both diagnostic and therapeutic purposes and can reduce pressure within the pancreatic duct, facilitating the closure of pancreatic fistulas. The closure rate of fistulas by ERCP is 55% in selected patients [6]. However, ERCP and interventions are not feasible in many cases of IPFs as traditional conservative management has been associated with prolonged hospitalization, malnutrition, and septic complications. Early surgical intervention, on the other hand, has been shown to treat both the fistula and the primary disease quickly, hence reducing morbidity risks associated with prolonged hospitalization [7].

## Materials and methods

This retrospective observational study was conducted between June 2018 and May 2020. Patients with acute or chronic pancreatitis and pleural effusion, ascites, and/or other IPFs were included in the study. The diagnosis of a pancreatic fistula was made based on fluid amylase levels >1,000 IU/L and fluid albumin levels >3 g/dL [7]. Patients with liver cirrhosis, suspected or proven malignancies, pleural effusion, or ascites that may be associated with heart disease, renal disease, or tuberculosis were excluded. Patients with previous pancreatic surgeries or iatrogenic injuries to the pancreas were also excluded.

**Figure 1:**
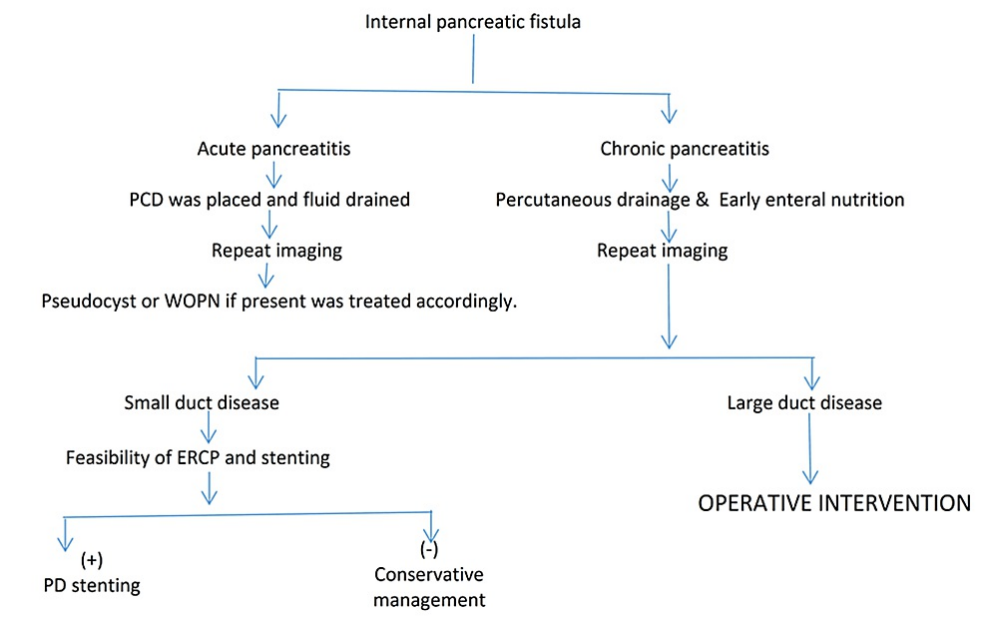
The treatment algorithm. WOPN: walled-off pancreatic necrosis; PCD: percutaneous catheter drainage; ERCP: endoscopic retrograde cholangiopancreatogram; PD: pancreatic duct

Patients suspected of having IPFs underwent the following assessments: complete blood counts, liver and renal function tests, and evaluation of blood sugar, C-reactive protein (CRP), serum amylase, and lipase levels. All patients underwent diagnostic paracentesis or thoracocentesis to confirm the diagnosis of pancreatic fistulas based on fluid amylase and albumin levels. The study group underwent ultrasonography and computed tomography (CT) of the abdomen and thorax, chest radiography, and magnetic resonance cholangiopancreatography (MRCP). These patients received treatment as proposed by the pre-planned algorithm as in Figure 1.

The traditional approach included expectant management with nil by mouth, parenteral nutrition, and octreotide. Failure to resolve at three weeks or progression and absence of symptomatic improvement were indications for escalation of treatment which included image-guided percutaneous drainage (PCD) or intercostal drainage. MRCP was obtained after PCD and before other interventions.

All participants followed accelerated treatment protocol which included PCD or ICD insertion on admission. At admission, patients were started on enteral feeding either orally or through a nasogastric/nasojejunal tube, octreotide, and albumin infusion. All patients were subjected to MRCP and a contrast-enhanced CT (CECT) of the abdomen and thorax. After complete drainage of the fluid, patients were taken up for the proposed intervention. The type of intervention was dependent on the acute or chronic nature, dilatation of the main pancreatic duct (MPD), and the presence of pseudocyst.

Those patients with acute pancreatitis were managed with expectant care. At the end of four weeks, patients were subjected to MRCP and CECT of the abdomen. The presence of pseudocyst or walled-off pancreatic necrosis was managed accordingly. Patients with chronic pancreatitis were divided into small duct disease (MPD <5 mm) and large duct disease (MPD >5 mm). Patients with small duct disease were evaluated by ERCP for the feasibility of sphincterotomy and/or pancreatic duct stenting.

The patients with large duct disease underwent upfront surgery. The procedures done were Frey’s procedure, Roux-en-Y lateral pancreaticojejunostomy, and distal pancreatectomy. All the patients were followed up for one year, and immediate postoperative complications and recurrence at one year were noted. Details of patients who underwent the early operative procedure are presented in Table 1.

**Table 1:** Characteristics of patients who underwent early surgical intervention.

Parameters studied	Early surgery group
Number	13
Mean age	42.8 years
Sex ratio	11:2
Mean serum amylase level	32 IU/L
Mean fluid amylase level	5,645 IU/L
Mean serum albumin	2.8 g/dL
Mean fluid albumin	3.2 g/dL

## Results

The study group consisted of 32 patients (27 male, five female) with IPFs, including pancreatic ascites, pancreatic pleural effusion, or both. Abdominal pain was the most frequent presenting symptom, and abdominal distention was a common clinical feature of pancreatic ascites. Patients with pancreatic effusion had few chest symptoms despite the presence of large amounts of fluid in the pleural cavity. Clinical features of acute pancreatitis were observed in 13 patients, while 19 had chronic pancreatitis. The mean duration of symptoms in patients with IPFs and acute pancreatitis was 8.4 days. Patients with chronic pancreatitis had symptoms lasting for more than one month (mean, 42 days). Associated pseudocysts and WOPN were also observed in 18 patients. The causative factors were alcohol consumption, gallstones, and tropical pancreatitis in 25, two, and five patients, respectively. Patient demographics are shown in Table 2.

**Table 2:** Patient demographics.

	Acute pancreatitis (n = 13)			Chronic pancreatitis (n = 19)		
Age (17–65 years)	Pleural effusion	Pancreatic ascites	Both	Pleural effusion	Pancreatic ascites	Both
Sex						
Male (27)	1	4	6	2	8	6
Female (5)	1	1	-	-	3	-
Symptoms						
Pain abdomen alone (11)	-	1	2	-	6	2
Pain abdomen + distention (8)	-	3	2	-	2	1
Abdominal distention alone (5)	-	2	-	-	3	-
Abdomen + chest symptoms (4)	1	-	1	-	-	2
Chest symptoms (5)	1	-	1	2	-	1
Etiology						
Alcohol	1	4	6	2	6	6
Gallstones	1	1	-	-	-	-
Tropical pancreatitis	-	-	-	-	5	-
Pseudocyst	-	4	3	1	6	4

In patients with acute pancreatitis-associated IPFs, a PCD catheter was placed for fluid drainage. Imaging was repeated after four weeks. If a pseudocyst or WOPN was present, it was treated accordingly. Pseudocysts were treated using laparoscopic cystoenteric drainage. Necrosis was treated with PCD, followed by video-assisted retroperitoneal debridement or cystoenteric drainage.

Patients with chronic pancreatitis were divided into those with small duct disease (MPD <5 mm), which was seen in six patients, and those with large duct disease (MPD ≥5 mm), which was seen in 13 patients. Patients with large duct disease underwent upfront surgery. Operative procedures included Roux-en-Y lateral pancreatojejunostomy, Frey’s procedure, and distal pancreatectomy (Table 3). The feasibility of performing ERCP and PD stenting in patients with small duct disease was evaluated. ERCP and stenting were attempted in six patients with small duct disease. However, stent implantation was successful in one patient. All other patients were managed conservatively. The mean duration of hospital stay in the early surgical intervention group was 13.6 days and in conservatively managed patients was 42.8 days. The total treatment duration for the remaining patients was 8-14 weeks. Postoperative complications observed included wound infection, grade A pancreatic fistulas, and pulmonary complications (Table 4). All complications were managed conservatively, and none required intervention or prolonged hospitalization.

**Table 3:** Operative procedures done in the early intervention group. LPJ: lateral pancreaticojejunostomy

Patient number	MRCP	Presentation	Duct size	Associated pseudocyst	Procedure done
1	Proximal disruption	Pancreatic ascites	9 mm	Yes	Frey’s procedure
2	Proximal disruption	Pancreatic ascites	7 mm	No	Frey’s procedure
3	Proximal disruption	Both	9 mm	Yes	Frey’s procedure
4	Proximal disruption	Pancreatic ascites	8 mm	Yes	Frey’s procedure
5	Proximal disruption	Pancreatic ascites	11 mm	No	Frey’s procedure
6	Proximal disruption	Pancraetic ascites	9 mm	Yes	Frey’s procedure
7	Proximal disruption	Both	10 mm	Yes	Frey’s procedure
8	Proximal disruption	Pancreatic ascites	11 mm	No	Frey’s procedure
9	Distal disruption	Pleural effusion	8 mm	No	Distal pancreatectomy
10	Distal disruption	Pleural effusion	6 mm	No	Distal pancreatectomy
11	Distal disruption	Both	6 mm	Yes	Distal pancreatectomy
12	Distal disruption	Pancreatic ascites	7 mm	Yes	Distal pancreatectomy
13	Indeterminate	Pancreatic ascites	8 mm	Yes	Roux-en-Y LPJ

**Table 4:** Complications noted in the early surgical group.

Parameters studied	All patients	Early surgery group (13)
Recurrence	5 (15%)	None
Wound infection	2 (6.25%)	2 (15.3%)
Hemorrhage	1 (3.1%)	None
External pancreatic fistula	4 (12.5%)	1 (grade A) (7.6%)
Sepsis	5 (15%)	0
Mean duration of hospital stay	36 days	13.6 days
Death	3 (9.3%)	None

Case one

A 37-year-old man presented to the emergency room with progressively worsening breathlessness for 10 days. The patient had been diagnosed with chronic pancreatitis six months prior during a regular follow-up. The patient was a known diabetic and was receiving regular treatment. On arrival, the patient had tachycardia with low pulse volume and tachypnea with normal room air saturation. Imaging showed a massive right-sided pleural effusion and mild peripancreatic collection. After initial resuscitation, a right chest tube was placed. The initial drain output was 2.5 L of brownish serosanguinous fluid. The pleural fluid amylase level was 11,500 U/L, and the albumin level was 3.2 g/dL.

**Figure 2:**
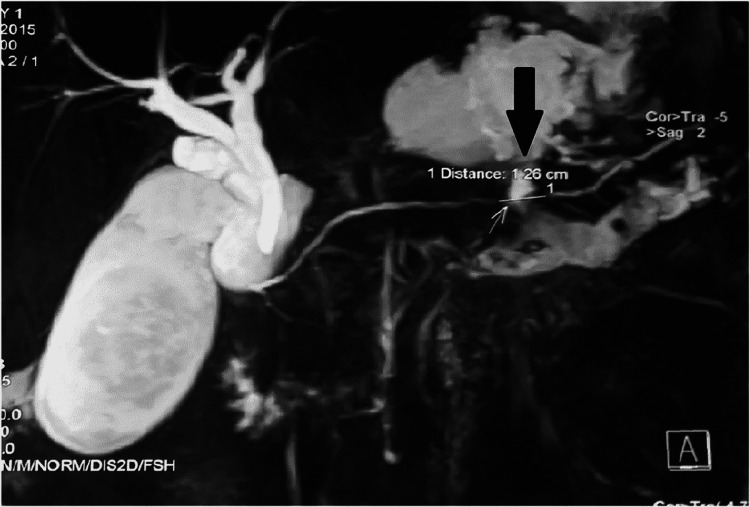
MRCP: disruption of the pancreatic duct in the tail of the pancreas with 3 × 2cm necrotic collection, which, in turn, was communicating with the right pleural cavity. MRCP: magnetic resonance cholangiopancreatography

MRCP revealed disruption of the pancreatic duct in the tail of the pancreas and a 3 × 2 cm necrotic collection, which was communicating with the right pleural cavity. MPD dilatation was 4 mm (Figure 2).

The patient was managed conservatively and improved symptomatically with gradual resolution of the right pleural fluid. The patient was started on an oral diet, which was well tolerated. ERCP was performed on day 12 of admission, and an attempt to cannulate the MPD was unsuccessful. The chest drain was removed on day 32, and a follow-up chest radiograph showed near-total resolution of pleural effusion.

Case two

A 20-year-old woman presented with upper abdominal pain for two months. On admission, her vital signs were normal. Abdominal examination revealed tenderness in the epigastric region and free fluid in the peritoneal cavity. The patient had elevated total leucocyte count (16,500 cells/mm^3^ of blood), serum amylase (820 IU/L), and lipase (269 IU/L) levels. CECT of the abdomen and pelvis revealed diffuse pancreatic calcifications with gross ascites. MRI of the abdomen showed features of chronic calcific pancreatitis and MPD dilatation of 4 mm.

The peritoneal fluid showed elevated amylase levels, confirming pancreatic ascites. Ultrasound-guided catheter drainage was performed. The patient continued to be symptomatic, with daily peritoneal drainage of >1 L for four weeks.

**Figure 3:**
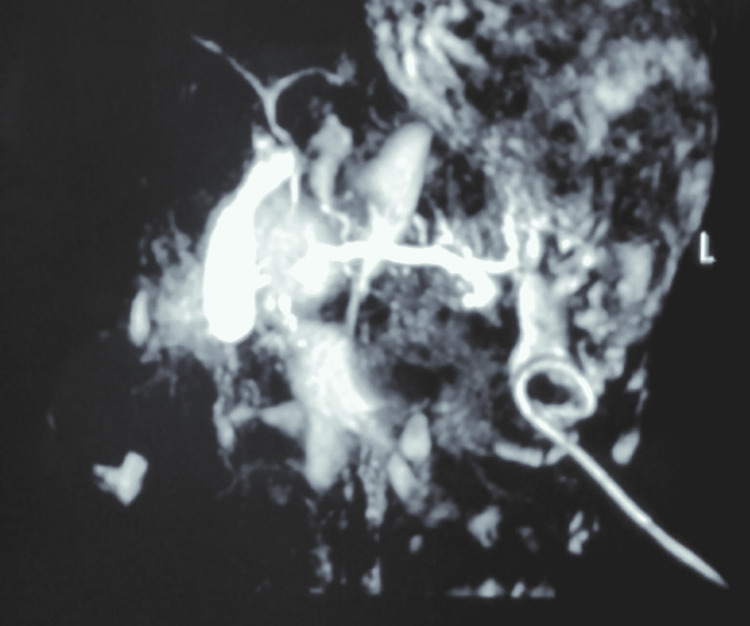
MPD disruption near the head of the pancreas with pigtail catheter in situ and resolving ascites. MPD: main pancreatic duct

MRCP revealed disruption of the MPD near the head of the pancreas with a pigtail catheter in situ and resolving ascites (Figure 3). ERCP revealed a leak in the pancreas, which was successfully stented using a 7-Fr 8 cm single pigtail stent. Free flow of pancreatic juice was noted. The patient was discharged and reviewed at two months and one year. The patient was asymptomatic and had no ascites.

Case three

A 52-year-old man with diabetes presented with abdominal pain and dyspnea for one week. The patient had been diagnosed with chronic pancreatitis one year previously. On admission, the patient had a normal hemodynamic status.

Systemic examination revealed ascites and a massive left-sided pleural effusion. Fluid analysis showed an elevated pleural fluid amylase level of 5,078 IU/L and an elevated ascitic fluid amylase level of 9,806 IU/L. The patient was treated with PCD of ascitic fluid and ICD tube drainage of the left pleural cavity.

**Figure 4:**
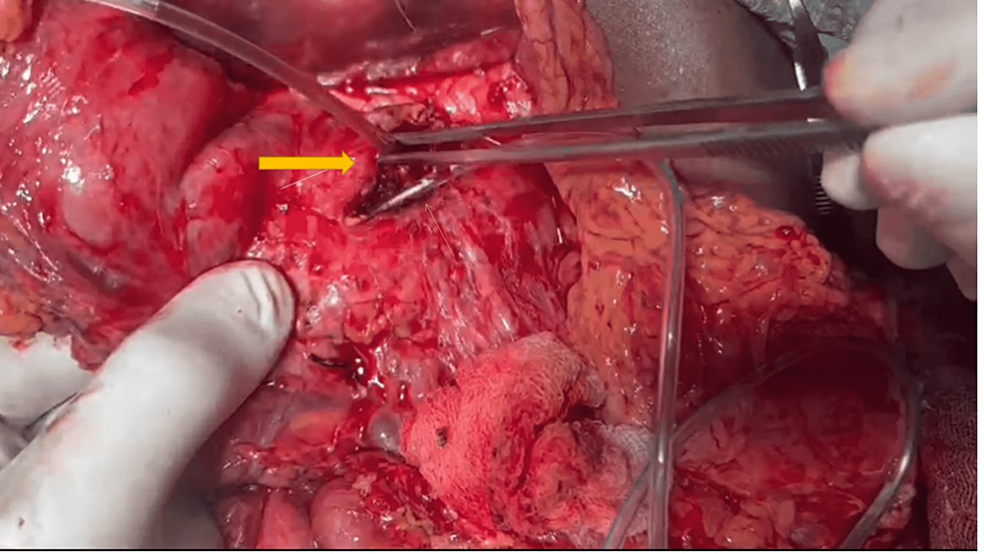
Intraoperative depiction: the white arrow shows the feeding tube entering the fistulous tract which is connected to MPD (yellow arrow) MPD: main pancreatic duct

The patient underwent MRCP, which revealed chronic calcific pancreatitis, MPD dilatation of 7 mm, and communication between the MPD and the left pleural cavity. The patient was scheduled for surgery on day four of admission. Peroperatively, there was dilatation of the MPD with multiple intraductal and parenchymal calcifications. A well-defined fistulous tract was noted between the MPD and the left pleural cavity (Figure 4). The patient underwent a lateral pancreaticojejunostomy. The patient was followed up for one year and had no recurrence of symptoms.

## Discussion

Pancreatic fistulas are rare disease entities that require a multidisciplinary approach. Pancreatic fistulas are classified in various ways. They can be classified anatomically as internal or external, or based on their fluid output as low-output (>200 mL) or high-output (<200 mL) fistulas [8]. They can also be classified etiologically as fistulas associated with acute pancreatitis, or fistulas associated with chronic pancreatitis. Lastly, they can be classified as simple (only one tract) or complex (more than one tract).

Common causes of IPFs are alcohol consumption, trauma, gallstone disease, hereditary pancreatitis, and autoimmune pancreatitis. The most common cause observed in our study was alcohol consumption, which was similar to findings from other studies. Tropical pancreatitis was observed in five patients and can be explained by the increased incidences of tropical pancreatitis in our region.

**Figure 5:**
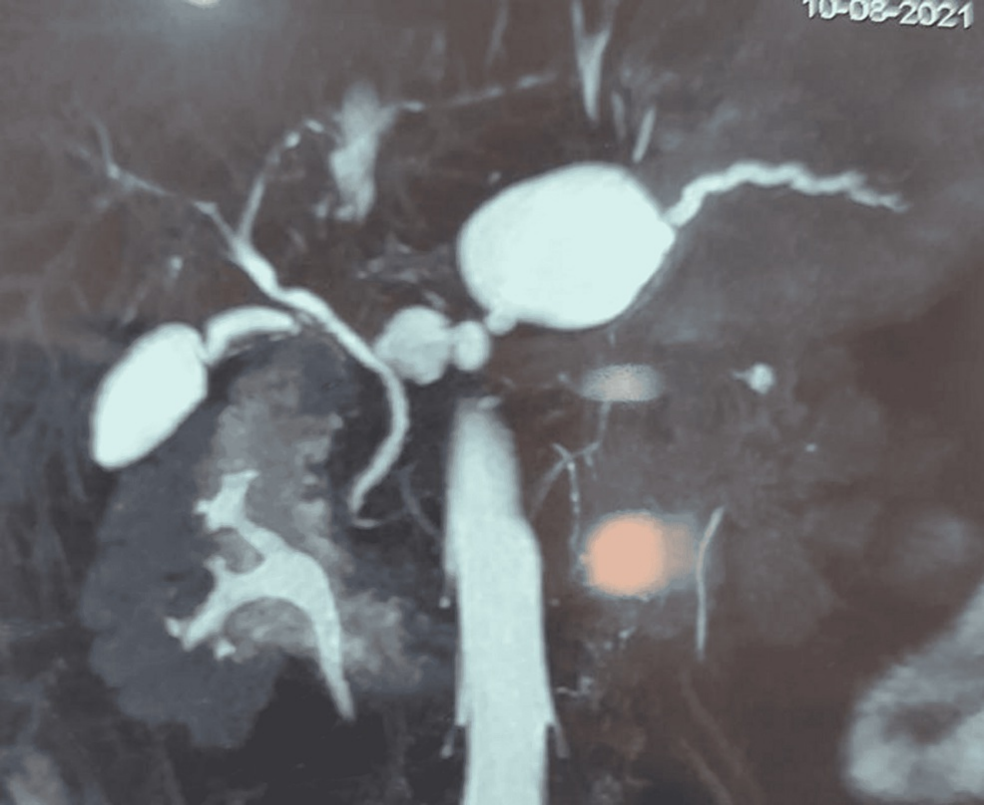
MRCP showing leaking pseudocyst with pancreatic ascites which was drained by PCD. MRCP: magnetic resonance cholangiopancreatography; PCD: percutaneous catheter drainage

The underlying mechanism of pancreatic fistulas due to any etiology is ductal disruption. Localized leaks can lead to the formation of a pseudocyst, whereas uncontained leaks can manifest as IPFs in the general peritoneal cavity, pleural cavity, bronchus, or mediastinum. Another mechanism of fistula formation is a leaking pseudocyst (Figure 5). In acute pancreatitis, ductal disruption is secondary to infected necrotic tissue or an ongoing inflammatory process. Ductal disruption of chronic pancreatitis is secondary to a leak caused by strictures or intraductal stones [9] with downstream obstruction [10]. They can be classified based on the output into low-output or high-output fistulas. Low output is less than 200 mL, and high output is more than 200 mL. Pancreatic fistulas can also be divided into simple (only one tract) or complex (more than one tract). Based on etiology, it can be classified as fistulas associated with acute pancreatitis or with chronic pancreatitis [10].

Clinical features can vary depending on the underlying etiology and type of IPF. Patient presentations can range from those who are asymptomatic to those who are severely ill with septic shock due to infection. Patients with pancreatic pleural effusion typically present with chest symptoms, such as dyspnea, cough, and chest pain, although the severity of symptoms does not always correlate with the severity of the disease. Pancreatic ascites can present as painless or with minimal abdominal pain [11].

The complications of pancreatic fistulas include nutritional deficiency, electrolyte imbalance, skin excoriation, infection, and, in rare cases, hemorrhage [11]. Pancreatic ascites do not cause peritonitis as ascitic fluid contains inactivated pancreatic enzymes. The enterokinase responsible for the activation of proenzymes is not present in the peritoneal or pleural cavity. If the fistula is associated with an enteric leak, the severity is increased due to the subsequent activation of pancreatic enzymes, which causes severe inflammation.

The diagnosis of pancreatic fistulas is based on fluid amylase >1,000 IU/L and fluid albumin >3 g/dL. Most patients in our study had higher serum amylase levels, which may be due to ongoing inflammatory processes in the pancreas or direct absorption of amylase from the peritoneal cavity. The cross-sectional imaging technique employed was CECT with MRCP. MRCP can help to delineate the fistulous tract, though the detection of the tract is not possible in all cases. In addition to the fistulous tract, MRCP can detect stones and strictures [12].

Traditional management includes conservative management after confirmation of the diagnosis [13,14]. If conservative management fails at the end of three weeks, a percutaneous drain is placed. Conservative management was successful in 30-60% of the patients. Some patients in our study required repeated exchange of drainage tubes due to catheter blocks or an inability to drain new collections. Patients underwent surgical management at the end of six weeks if the intervention failed. Due to prolonged hospitalization and loss of proteins through drain tubes, these patients suffered from malnutrition and electrolyte imbalance.

In our study, patients were initially evaluated for ERCP and endoscopic stenting. In most patients, stenting was either not feasible or failed. Only one patient was successfully treated. In a similar study by Wronski et al., approximately half of the patients were successfully stented [15].

Both the fistula and underlying pathology were simultaneously treated in patients with chronic pancreatitis who underwent early surgery. Early surgical intervention is associated with morbidity rates of 12% and mortality of 2-3% [16]. In a study by Dhali et al., alcohol was the most common etiology, as seen in our study, and pseudocyst was a common association. Lateral pancreaticojejunostomy was done in 11 patients. Similar to our study, early surgical intervention reduced hospital stays and rates of complications. Endoscopic interventions were attempted in six patients and were unsuccessful [17].

In our study, complications, including surgical site infections, were low in the early surgical group. The duration of hospital stay was 8-14 weeks in the traditional intervention group compared to 8-10 days in the early surgical intervention group. The main complications observed across the entire study group were recurrence in 15% of patients, septic complications in 15%, and postoperative pancreatic fistulas (POPFs) in four patients. All POPFs were grade A and treated conservatively. In the early surgical intervention group, wound infection was seen in two patients and POPF grade A in one patient. No mortality was observed in the surgical intervention group. However, two deaths were observed among the rest of the patients.

Early surgical intervention was associated with lower recurrence rates, morbidity rates, and cost. Furthermore, it resulted in faster resolution of symptoms and addressed the primary etiology.

## Conclusions

IPFs are associated with low overall mortality rates but high morbidity rates. Delay in treatment may be a contributing factor to high morbidity rates; hence, early surgical intervention may be key in changing the clinical course and addressing the primary pathology of the pancreas. However, the benefits and complications of traditional management and surgical intervention should be further evaluated through randomized control studies to develop a standardized protocol.
